# Decolonizing Global Health: Increasing Capacity of Community Health Worker Programs

**DOI:** 10.5334/aogh.4325

**Published:** 2023-12-22

**Authors:** Pamela Avila

**Affiliations:** 1 Legacy Health System/Oregon Health & Science University, US

**Keywords:** community health workers, decolonization of global health, global medical volunteers

## Abstract

Many global health volunteer experiences and research projects are focused on the needs of the host country participants, which perpetuates a sovereign or superior relationship towards low- or middle-income countries (LMIC). The purpose of this paper is to discuss ethical and culturally sensitive practices in LMIC when providing health care as a volunteer or researcher. International ethical standards for providing global health care are discussed. The author participated in a volunteer global health experience for three months in Kenya. An evaluation of a nongovernmental organization (NGO)-sponsored community health worker (CHW) program was conducted and is presented here. Health indicators such as prenatal care visits, birth attendance by skilled personnel, and full vaccination of children improved by 34%, 36%, and 24%, respectively, following 5 years of implementation of the CHW program. Global health care can be provided in a more socially responsible and sustainable manner by supporting NGO-sponsored CHW programs affiliated with local ministries of health in LMICs.

## I. Introduction

Awareness of global health issues is growing among primary care providers, health care students, and educators. As more immigrant and refugee populations settle in the US, it is essential that health care providers understand the social and medical issues affecting these populations. While experiential global health programs can be life-changing for the participants, volunteer or research programs are often focused on the desires of institutions in high-income countries (HIC), not those of hosts, which are usually low- or middle-income countries (LMIC). Typically, the goal of HIC medical institutions is not simply to provide humanitarian aid but to conduct research or educate health care students. This includes providing the host country with the research proposal and study design and presenting and publishing the findings in English-language journals or conferences that may be inaccessible to LMIC partners. Where global health nongovernmental organizations (NGO) may focus solely on providing humanitarian health care, missions are generally short-term and may perform surgeries without the benefit of follow-up care. Often, the goal is to create learning experiences for nursing or medical students. Many global health NGOs are faith-based organizations whose secondary agenda may include evangelization. Additionally, donations may include medical equipment (which might be difficult for LMIC to maintain) or long-term medications that are likely to expire or run out soon after a short supply is given to the patient. Historically, many Asian, African, and Latin American countries suffered abuse by European nations that exploited resources for their own gain. Institutions providing humanitarian aid need to avoid practices that might be reminiscent of LMIC colonization by HIC [2]. Likewise, any practices that initiate services without a clear follow-up plan would be considered unsustainable (such as providing primary care in a LMIC for a week without referrals to local in-country health care providers).

A statement of ethical principles was created in 2020 known as the Brocher Declaration, which provided guidelines for short-term global health engagements (including clinical and other educational exchanges, student research projects, and medical mission trips). Principles include prioritizing mutual respect, focusing on sustainable programming and capacity building, and complying with applicable laws and ethical standards [1].

It is therefore essential that all global health programs and educational institutions consider engaging in a different model of humanitarian aid, principally centered on teaching, mentoring, or providing technical assistance to LMIC health care workers who are integrated within their own public health system [4]. By this approach, the host country takes the lead in requesting the assistance most needed by the local population, and the project is culturally sensitive, which helps to “decolonize” global health [3]. One way of decolonizing global health is by supporting existing community health worker programs in LMICs. In this way, continuity of care is more likely to occur once the foreign volunteers have gone home.

## II. Community Health Worker Programs: Background Information

The development of community health worker programs began during the late 20^th^ century in an attempt to expand health care coverage in LMICs. In 2015, responding to a need for more structured global development, the United Nations created the Sustainable Development Goals (SDGs). The SDGs are designed to improve access to safe drinking water and sanitation, reduce poverty and hunger, empower marginalized populations, and protect the planet. The goals, which hope to achieve health-related targets by 2030, replaced the Millennium Development Goals and are more internationally inclusive, acknowledging greater HIC responsibility.

Compared with HIC, where there are an average of 3.2 physicians per 1,000 people, countries in sub-Saharan Africa have an average of 0.2. The global shortage of licensed health care providers is currently between 4 and 6 million, mostly in south Asia and Africa [9]. Because of this shortage, health care is often provided by community health workers, who offer health education, screen for disease, and refer the most seriously ill individuals to local health care facilities. Among the advantages of these programs are:

1. Community health workers (CHWs) are residents of the villages they are assigned to and are known and respected by their clients
2. CHWs are familiar with local cultural practices
3. CHWs operate under specific protocols and do not need to have formal medical education
4. CHWs produce a great return on investment

Evidence exists that CHW programs are effective in providing reproductive health services, maternal and newborn health services, child health services, communicable and noncommunicable disease prevention and treatment, and mental health services. The CHW model has proven to be a sustainable and practical solution to increasing health care access in LIC, which is essential to quality of life, as countries with inadequate public health systems are associated with poor economic growth and political instability [5].

The World Health Organization (WHO) has published guidelines that direct government institutions and NGOs on the training and support of CHWs. According to these guidelines, areas of focus should include:

1. Selection criteria that specify minimum education
2. Pre-service training with a competency-based curriculum
3. Hands-on training with certificate provision upon completion
4. Supportive supervision with regular performance evaluation
5. Remuneration with potential career advancement
6. Integration into existing health systems that take into account population size
7. Utilization of data collection
8. Engagement of community representatives in planning
9. Assurance of an adequate supply chain for CHWs to accomplish their work [10]

CHW motivation can be affected by several intrinsic and extrinsic factors, such as financial and in-kind material compensation. Paid CHWs usually have more motivation, productivity, and retention. These factors are affected, however, by expectations to be paid regularly at a particular rate or by the quality of other material incentives. Material incentives might include T-shirts, bicycles, mobile phones, and other job aids, as well as opportunities for advancement and further education [7].

Best practices for successful CHW programs include ensuring integration and standardization within existing Ministry of Health systems. This approach identifies local government goals and funding (rather than being project-focused and based on NGO interests) and is unified throughout the country (not limited to particular regions). Quality CHW programs are inclusive of all health care needs for the population served [8].

The supervision of CHWs is key to overcoming challenges to program success. Many issues result from a chain of events, and quality improvement requires systems analysis. Management has to be adaptable, and a team approach is necessary, rather than simply assigning blame to individuals who are seen as performing poorly. Planning and re-evaluation need to be continuous and must involve all stakeholders, with changes based on data gathered during project implementation. In this way, problems arising from inadequate leadership, limited government engagement, issues related to the material supply chain, malfunctioning equipment, and financing can be properly addressed [8].

## III. Model CHW Program in Kenya

An example of a successful CHW program is one operated by the NGO Catholic Medical Mission Board (CMMB) in South Kitui County, Kenya. (In Kenya, they are known as Community Health Volunteers or Community Health Promoters, acknowledging the fact that they are not salaried workers, though for simplicity, they will continue to be referred to here as CHWs.) The main focus is improving health outcomes for mothers and children. Interventions are carried out by a group of CHWs who are assigned to particular communities and are supervised by lead CHWs, who in turn report to government-run health care facility staff. Among the tasks that CHWs perform, they may assure that:

1. Pregnant women have at least 4 antenatal visits
2. Women deliver in hospitals with skilled birth attendants and receive postpartum care
3. Infants receive health care during the neonatal period
4. Children under five are assessed and treated for malnutrition
5. Children under five are assessed and referred for illnesses such as pneumonia and diarrhea
6. Children are fully immunized

CMMB supervises around 350 CHWs in south Kitui County, who each have a caseload of between 50 and 200 households. CHWs are recruited continuously from the local villages. CMMB staff provide new volunteers with 80 hours of initial training, including didactic and practical components. Regularly scheduled meetings (driven by less-than-optimal outcomes in particular program sectors) are held monthly, which include a review of data, a discussion of challenges experienced by CHWs, and a health care skills review. Lunch is generally provided, and CHWs have an opportunity to network with peers and CMMB staff. Mentoring, dealing with stock-outs, and bringing pilot projects up to scale are common issues that arise in the supervision of CHWs. For example, if neonatal deaths are trending higher, staff will meet the CHW in that community to determine whether pregnant women and newborns have attended health care appointments. Likewise, if the assessment of pneumonia is affected by the fact that none of the CHWs’ thermometers are working, this supply chain problem will be addressed. CHWs are expected to keep paper and mobile cellular phone records of household visits and outcomes, which are incorporated into the electronic Kenya Health Information System. Quality assurance audits are routinely conducted at hospital and clinic facilities to determine the availability of supplies, assess the existence and function of infrastructure (water, electricity), and determine whether staff are properly trained. Inadequacies are addressed by the Ministry of Health at both implementation and policy levels based on data collection, which is utilized to determine budget and funding.

**Figure 1:**
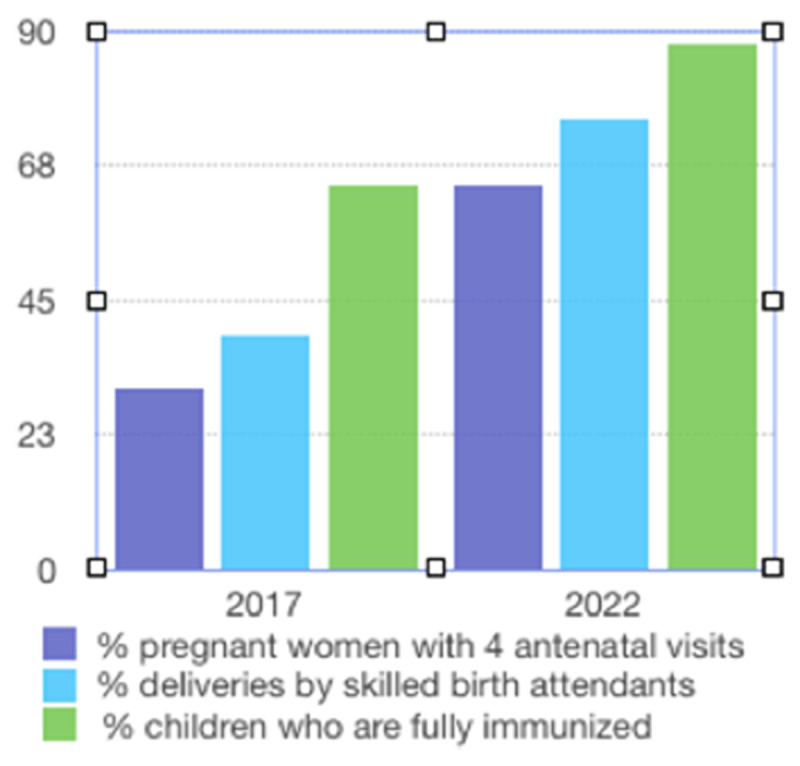
Improvement in Health Indicators South Kitui County, Kenya. *Data Obtained from Kenya Health Information Services.

In the five-year period of 2017–2022, health indicators have demonstrated significant improvements in south Kitui County, based on the CMMB-sponsored CHW program, as noted in Figure 1. Health Indicator Improvement in south Kitui County. For example, during this time period, antenatal visits increased from 30 to 64%, deliveries by skilled birth attendants increased from 35 to 75%, and childhood immunization increased from 64 to 88% [6].

## IV. Discussion

The author spent three months volunteering under the auspices of CMMB. In addition to accompanying Kenyan colleagues in their supervision of CHWs, the author had opportunities to teach nursing students, conduct continuing medical education training for hospital staff, and participate in a gender-based violence committee. In the course of accomplishing this work, the author was confronted with many cultural challenges related to how meetings were run, how students were taught, technical and infrastructure problems, procurement of supplies, and expectations about government involvement. It was a rich and memorable experience, with the focus being more on intercultural collaboration and less on technical contributions made by one volunteer.

The principal goal of global health volunteers should be to increase the capacity of the host country’s health care workforce. Work that is done in collaboration with the local Ministry of Health can be supported by international volunteers, whose technical, medical, educational, and engineering skills can make significant contributions as guest instructors and mentors/consultants to existing local personnel. The most successful programs credit their success to the relationships that are established between local and international partners. These relationships typically require time to develop, therefore the longer a volunteer is committed to service, the stronger the impact will be. This is not usually accomplished on a week-long medical mission trip and should be considered when selecting or establishing experiences for professionals or students. When global health work is collaborative, it becomes more sustainable. Ideally, skills and resources would be transferred to the local workforce. Volunteers should essentially “work their way out of the job” and not perform tasks that local community members are capable of doing themselves.

The benefits of global health experiences for volunteer participants are invaluable. In preparation for the medical service trip, volunteers can raise awareness about the needs of the population they will be serving (as well as funds). Many returned volunteers will have new insight into limiting consumption and a desire to disseminate information about their experience. This, in turn, improves the health care provider’s ability to serve local immigrant and refugee populations in their home country. When sustainable health care is provided in partnership over a committed period of time, the learning is mutual, barriers are broken, and new levels of understanding are achieved, which, in the end, are the goals of many global health programs.
